# Antibody-Dependent Dengue Virus Entry Modulates Cell Intrinsic Responses for Enhanced Infection

**DOI:** 10.1128/mSphere.00528-19

**Published:** 2019-09-18

**Authors:** Candice Y. Y. Chan, John Zhong Heng Low, Esther Shuyi Gan, Eugenia Ziying Ong, Summer Li-Xin Zhang, Hwee Cheng Tan, Xiaoran Chai, Sujoy Ghosh, Eng Eong Ooi, Kuan Rong Chan

**Affiliations:** a Program in Emerging Infectious Diseases, Duke-NUS Medical School, Singapore; b Department of Infectious Diseases, Singapore General Hospital, Singapore; c Program in Cardiovascular & Metabolic Disorders, Duke-NUS Medical School, Singapore; d Centre for Computational Biology, Duke-NUS Medical School, Singapore; e Department of Microbiology and Immunology, Yong Loo Lin School of Medicine, National University of Singapore, Singapore; f Saw Swee Hock School of Public Health, National University of Singapore, Singapore; University of Pittsburgh School of Medicine

**Keywords:** dengue virus, host response, virus-host interactions

## Abstract

Dengue is caused by infection with any one of four dengue viruses (DENV); the risk of severe disease appears to be enhanced by the cross-reactive or subneutralizing levels of antibody from a prior DENV infection. These antibodies opsonize DENV entry through the activating Fc gamma receptors (FcγR), instead of infection through canonical receptor-mediated endocytosis, to result in higher levels of DENV replication. However, whether the enhanced replication is solely due to more efficient FcγR-mediated DENV entry or is also through FcγR-mediated alteration of the host transcriptome response to favor DENV infection remains unclear. Indeed, more efficient viral entry through activation of the FcγR can result in an increased viral antigenic load within target cells and confound direct comparisons of the host transcriptome response under antibody-dependent and antibody-independent conditions. Herein, we show that, despite controlling for the viral antigenic load in primary monocytes, the antibody-dependent and non-antibody-dependent routes of DENV entry induce transcriptome responses that are remarkably different. Notably, antibody-dependent DENV entry upregulated DENV host dependency factors associated with RNA splicing, mitochondrial respiratory chain complexes, and vesicle trafficking. Additionally, supporting findings from other studies, antibody-dependent DENV entry impeded the downregulation of ribosomal genes caused by canonical receptor-mediated endocytosis to increase viral translation. Collectively, our findings support the notion that antibody-dependent DENV entry alters host responses that support the viral life cycle and that host responses to DENV need to be defined in the context of its entry pathway.

**IMPORTANCE** Dengue virus is the most prevalent mosquito-borne viral infection globally, resulting in variable manifestations ranging from asymptomatic viremia to life-threatening shock and multiorgan failure. Previous studies have indicated that the risk of severe dengue in humans can be increased by a specific range of preexisting anti-dengue virus antibody titers, a phenomenon termed antibody-dependent enhancement. There is hence a need to understand how antibodies augment dengue virus infection compared to the alternative canonical receptor-mediated viral entry route. Herein, we show that, besides facilitating viral uptake, antibody-mediated entry increases the expression of early host dependency factors to promote viral infection; these factors include RNA splicing, mitochondrial respiratory chain complexes, vesicle trafficking, and ribosomal genes. These findings will enhance our understanding of how differences in entry pathways can affect host responses and offer opportunities to design therapeutics that can specifically inhibit antibody-dependent enhancement of dengue virus infection.

## Abstract (precis)

Dengue virus is the most prevalent mosquito-borne viral infection globally, resulting in variable manifestations ranging from asymptomatic viremia to life-threatening shock and multiorgan failure. Previous studies have indicated that the risk of severe dengue in humans can be increased by a specific range of preexisting anti-dengue virus antibody titers, a phenomenon termed antibody-dependent enhancement. There is hence a need to understand how antibodies augment dengue virus infection compared to the alternative canonical receptor-mediated viral entry route. Herein, we show that, besides facilitating viral uptake, antibody-mediated entry increases the expression of early host dependency factors to promote viral infection; these factors include RNA splicing, mitochondrial respiratory chain complexes, vesicle trafficking, and ribosomal genes. These findings will enhance our understanding of how differences in entry pathways can affect host responses and offer opportunities to design therapeutics that can specifically inhibit antibody-dependent enhancement of dengue virus infection.

## INTRODUCTION

Dengue is the most prevalent arthropod-borne viral disease globally. Infection with any one of four antigenically different dengue viruses (DENVs) can result in a range of outcomes, from asymptomatic infection to severe dengue with life-threatening hypovolemic shock due to vascular leakage, internal hemorrhage, and multiorgan dysfunction (1). The risk of severe disease is increased during secondary infection with a DENV that is heterologous in type compared to the type responsible for the primary infection. It has long been postulated through epidemiological observations and cohort studies that cross-reactive and nonneutralizing or subneutralizing levels of antibodies that develop following a primary DENV or flaviviral infection can bind to heterologous DENV to enhance infection, therefore resulting in a high viral burden and a strong disease severity (2–10). This postulate has been aptly termed antibody-dependent enhancement (ADE) of DENV infection. In a prospective study in Bangkok, Thailand, serum specimens were collected from schoolchildren who developed severe secondary dengue and tested for their ability to enhance DENV type 2 (DENV2) replication in human monocytes *in vitro* (11). The results from that study indicated that the specific titers of nonneutralizing dengue virus antibodies can be enhancing and are associated with an increased risk of severe dengue disease. Indeed, a long-term observation of a pediatric cohort in Nicaragua confirmed that ADE of dengue disease occurred in human subjects with presecondary infection anti-DENV antibody at a specific range of concentrations (12). Likewise, a school-based cohort study in Thailand demonstrated that preexisting heterologous anti-DENV antibodies with low hemagglutination inhibition assay titers were associated with the subsequent development of severe disease (13). Finally, a clinical trial using a live attenuated vaccine against yellow fever virus, also a flavivirus related to DENV, showed that subjects with cross-reactive antibodies within a specific range of titers produced longer-lasting vaccine viremia and correspondingly higher yellow fever virus neutralizing antibody titers than those without cross-reactive antibodies (14). Collectively, these findings support the notion that ADE is an important pathogenic factor in dengue.

Despite the association between ADE and severe dengue, the mechanism by which antibodies augment DENV infection remains incompletely understood. Early studies elucidated that subneutralizing levels of antibodies can facilitate virus entry by forming immune complexes that interact with activating Fc gamma receptors (FcγR) that are expressed on myeloid-derived cells, such as monocytes, macrophages, and dendritic cells (3, 4, 15–18). In contrast, under non-ADE conditions, DENV infects the host cell via canonical receptor-mediated endocytosis (19). Besides the differential usage of receptors, the internalization of viral particles after FcγR- and DENV receptor-mediated entry is distinguished by the different utilization of clathrin, actin, phosphatidylinositol 3-kinase, and Rab GTPases (20, 21), which may consequently result in differences in virus compartmentalization (22). Receptor usage and viral entry pathway differences are thought to alter the host intrinsic environment that supports the viral life cycle, including viral compartmentalization, fusion, replication, and translation (4, 14, 22–27). Indeed, by inoculating monocytes with DENV at a multiplicity of infection (MOI) that matched the antigenic load between non-ADE (DENV only) and ADE conditions, we and others have previously demonstrated that ADE-mediated infection produces more infectious virions than infection with DENV only (24, 25, 28, 29). While the suppression of innate cellular antiviral responses at the later stages of infection under ADE conditions could be one factor contributing to the promotion of viral infection, the extent to which genes are regulated by differential receptor engagement and signaling during infection is unclear, partly because DENV infection of monocytic cells is significantly more efficient under ADE conditions (30). However, previous studies analyzed the entire cell culture population as a whole and did not take into consideration the heterogeneity in viral uptake (31, 32), which can itself result in variability in gene expression, thus making it difficult to identify early gene expression changes that are mediated by different routes of entry among cells with equivalent antigenic loads.

Here, we compared the transcriptome of ADE-mediated infection and infection with DENV only in primary monocytes. We coupled Alexa Fluor-labeled DENV with cell sorting to derive cells with similar antigenic loads despite different entry routes. We found that the route of entry impacted early the transcriptional response to DENV. Specifically, we identified that ADE significantly induced genes associated with RNA splicing, mitochondrial respiratory chain complexes, and vesicle trafficking. Antibody-mediated DENV entry also impeded the downregulation of ribosomal genes caused by canonical receptor-mediated endocytosis. In addition, by cross-referencing our data with existing data sets on the host dependency factors of flaviviruses (33–37), we uncovered a list of viral dependency genes that are uniquely upregulated during ADE-mediated DENV infection. Our findings reveal how ADE alters intrinsic host responses to promote virus infection and offers opportunities to design therapeutics that can manipulate these critical host factors.

## RESULTS

### Balancing the viral antigenic load in primary monocytes despite different routes of viral entry.

**FIG 1:**
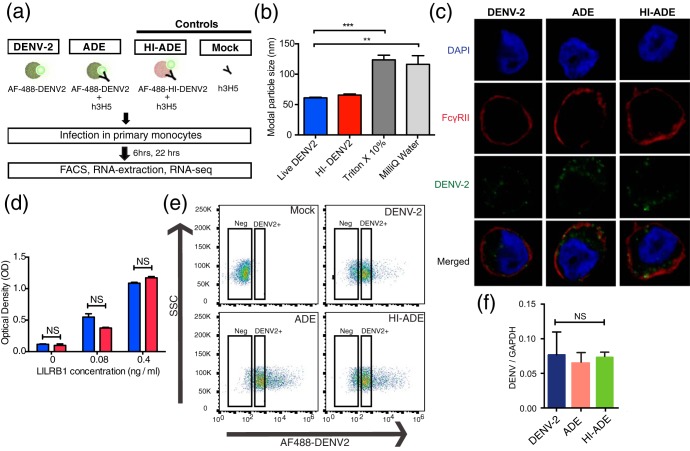
Experimental strategy to isolate infected monocytes with a similar antigenic load. (a) Schematic diagram of the experimental design. Primary human monocytes were infected with Alexa Fluor 488-labeled DENV2 (AF488-DENV2) or AF488-DENV2 opsonized with h3H5 at an MOI of 10. Heat-inactivated AF488-DENV opsonized with h3H5 (HI-ADE) and h3H5 treatment (mock infection [Mock]) served as controls. After 6 and 22 hpi, infected monocytes with an equivalent antigenic load were sorted by FACS and RNA was extracted for RNAseq. (b) Modal particle size of DENV2 and inactivated DENV2 treated with heat (heat-inactivated DENV2 [HI-DENV2]), Triton X-100, or Milli-Q water, determined using nanoparticle tracking analysis. (c) Localization of AF488-DENV2 in THP-1 cells under DENV2, ADE, or HI-ADE conditions after 6 hpi. DAPI is represented in blue, FcγRII is represented in red, and AF488-DENV2 is represented in green. (d) Binding of DENV2 and HI-DENV2 to LILRB1, determined by ELISA. (e) Representative flow cytometry plots for conditions of mock, DENV2, ADE, and HI-ADE infection after 6 hpi. Cells within a restricted range of fluorescence intensity (DENV2^+^) were sorted by FACS for further transcriptomic analysis. All subsequent experiments and analyses were performed on the DENV2^+^ cell population. SSC, side scatter. (f) Viral RNA copy numbers of the DENV2^+^ cells under DENV2, ADE, and HI-ADE conditions after 6 hpi. Data are represented as the mean ± SD from three independent experiments. Experiments were performed in three replicates. NS, not significant.

To identify host gene expression changes mediated by different entry routes under DENV and ADE conditions while controlling for viral antigen load, we infected primary monocytes with Alexa Fluor 488 (AF488)-labeled DENV (AF488-DENV) only (38) or AF488-DENV opsonized with subneutralizing levels of chimeric human/mouse IgG1 3H5 (h3H5) monoclonal antibody. After 6 and 22 h postinfection (hpi), fluorescence-activated cell sorting (FACS) was used to sort for monocytes with a specific range of fluorescence intensities (Fig. 1a). Cells infected with h3H5 only (mock-infected cells) and heat-inactivated DENV2 opsonized with h3H5 (HI-ADE) were used as controls for comparison; the use of HI-ADE enabled us to distinguish the transcriptomic changes induced by receptor ligation from those influenced by differential trafficking or compartmentalization of DENV. We also ascertained that heat inactivation did not cause the formation of large viral aggregates (Fig. 1b) that would have bound the inhibitory FcγRIIB expressed at low levels to inhibit phagocytosis (Fig. 1c) or its interaction with the leukocyte immunoglobulin-like receptor B1 (LILRB1) (Fig. 1d), both of which have previously been shown by us to impact ADE (20, 32). This experimental setup thus allowed us to distinguish the gene expression changes triggered by the respective viral entry pathway independently of the antigen load.

Consistent with the ADE phenomenon, AF488-DENV2 opsonized with a subneutralizing concentration of h3H5 augmented viral entry compared to that achieved with DENV2 alone, as evidenced by an increased percentage of DENV2-positive (DENV2^+^) cells and a higher mean fluorescence intensity (MFI) after 6 hpi (Fig. 1e) and 22 hpi (see Fig. S1a in the supplemental material), respectively. To obtain cells with similar viral antigenic loads, we sorted a specific gate of DENV2^+^ cells under ADE, DENV, and HI-ADE conditions with an equivalent MFI. Verification of the equivalence of the antigenic loads was also carried out by reverse transcription (RT)-quantitative PCR (qPCR), which showed similar numbers of viral RNA copies in the sorted cells (Fig. 1f). However, at 22 hpi, the degradation of viral RNA was observed under HI-ADE conditions (Fig. S1b).

10.1128/mSphere.00528-19.1 FIG S1 The use of FACS to isolate infected cells with a similar antigenic load. (a) Representative flow cytometry plots for mock-infected, DENV2, ADE, and HI-ADE conditions after 22 h postinfection (hpi). Cells within a restricted range of fluorescence intensity (DENV2^+^) were sorted by FACS for further transcriptomic analysis. (b) Viral RNA copy numbers of the DENV2^+^ cells under DENV2, ADE, and HI-ADE conditions after 22 hpi. The data are represented as the mean ± SD from three independent experiments. Experiments were performed in three replicates. Download FIG S1, TIF file, 2.2 MB. Copyright © 2019 Chan et al. 2019 Chan et al. This content is distributed under the terms of the Creative Commons Attribution 4.0 International license.

### ADE induced transcriptional changes distinct from those induced by canonical receptor-mediated viral entry.

We first utilized sequencing with a MiSeq sequencer to gain preliminary insights into the transcriptome landscape of the antigenically balanced cells under ADE, DENV, and HI-ADE conditions after 6 and 22 hpi. All gene expression changes were normalized to the levels of expression by mock-infected cells, and genes with *P* values of <0.05, a false discovery rate (FDR) of ≤0.1, and a fold change in expression of at least ≥1.5 were considered differentially expressed genes (DEGs). Gene expression changes were detected at both 6 and 22 hpi, with a greater number of differences being detected at 6 hpi than at 22 hpi (Fig. S2a). Only a minor subset of genes overlapped between DENV, ADE, and HI-ADE conditions, suggesting that the different routes of viral entry resulted in unique transcriptional profiles (Fig. S2a).

10.1128/mSphere.00528-19.2 FIG S2 Distinct gene profiles of the infected monocytes under DENV, ADE, or HI-ADE conditions at 6 and 22 h postinfection. (a) Venn diagram showing the number of differentially expressed genes that are upregulated (left) and downregulated (right) after 22 hpi under DENV, ADE, and HI-ADE conditions, identified with the MiSeq platform. (b) Correlation between MiSeq and HiSeq reads at 6 hpi based on FPKM values. The Pearson correlation values (*R*) for the respective conditions are displayed, with the *P* value being ≤0.001 for all conditions. Download FIG S2, EPS file, 2.8 MB. Copyright © 2019 Chan et al. 2019 Chan et al. This content is distributed under the terms of the Creative Commons Attribution 4.0 International license.

**FIG 2:**
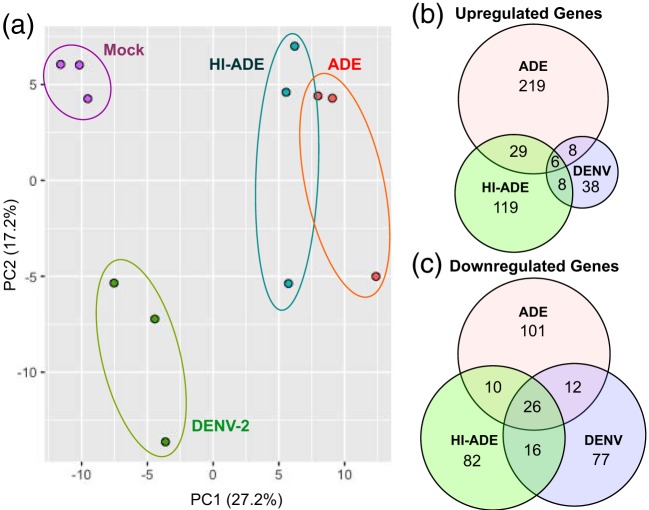
Distinct gene profiles of monocytes under DENV, ADE, or HI-ADE conditions. (a) Principal-component analysis (PCA) of RNAseq data from mock-infected and DENV2^+^ primary monocytes obtained from the assay whose results are presented in Fig. 1e infected under ADE, DENV, and HI-ADE conditions. (b and c) Venn diagrams showing the number of upregulated (b) and downregulated (c) differentially expressed genes (DEGs) after 6 hpi under DENV, ADE, and HI-ADE conditions, identified with the HiSeq platform. All DEGs have *P* values of <0.05, a false discovery rate (FDR) of ≤0.1, and a fold change in expression of at least ≥1.5 compared to the level of expression by mock-infected cells, based on three independent experiments.

To obtain a more detailed map of the transcriptomic differences, we next subjected the RNA extracted from sorted cells to sequencing with a HiSeq sequencer. We observed a high concordance between the data obtained with the MiSeq and HiSeq sequencers (Fig. S2b). After preprocessing and normalization of the HiSeq sequencing data, unsupervised analysis using principal-component analysis (PCA) was used to visualize the relationship between mock-infected, ADE, DENV, and HI-ADE conditions. PCA indicated that the route of viral entry was the primary cause of the transcriptional changes, as shown along principal component 1 (PC1) (Fig. 2a). The comparison of different infection conditions and uninfected samples at 6 hpi identified 262, 60, and 162 significantly upregulated DEGs under ADE, DENV, and HI-ADE conditions, respectively (Fig. 2b). Conversely, 149, 131, and 134 DEGs were downregulated under these respective conditions (Fig. 2c). The identities and expression levels of these DEGs are summarized in Table S1. Consistent with the MiSeq sequencing and PCA analyses, a low degree of overlap was observed between the different infection conditions (Fig. 2b and c), which supports the notion that ADE-mediated viral entry in monocytes alters a set of genes unique from the sets of genes seen to be altered under DENV and HI-ADE conditions.

10.1128/mSphere.00528-19.6 TABLE S1 Raw data set reflecting transcriptomic changes under DENV2, ADE, and HI-ADE conditions with respect to expression under mock-infected conditions. Download Table S1, XLSX file, 0.05 MB. Copyright © 2019 Chan et al. 2019 Chan et al. This content is distributed under the terms of the Creative Commons Attribution 4.0 International license.

### ADE upregulated genes involved in RNA processing and mitochondrial respiratory chain complexes.

**FIG 3:**
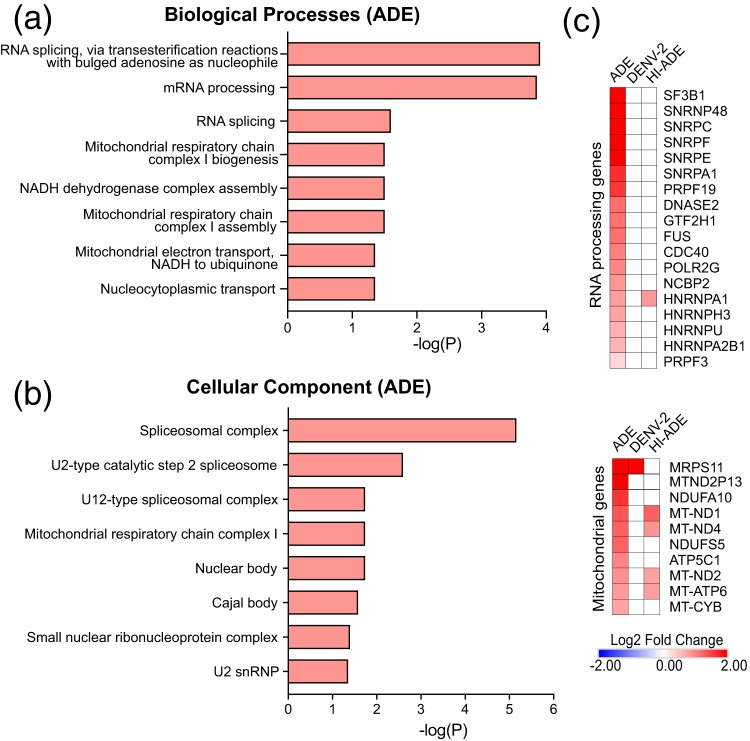
ADE-mediated DENV entry upregulates the expression of genes related to RNA processing and mitochondrial respiratory chain complexes. (a and b) Gene Ontology (GO) annotations of differentially expressed genes (DEGs) at 6 hpi after ADE-mediated DENV viral entry, classified by biological processes (a) and cellular component (b), as determined using EnrichR analysis. Red bars indicate significantly increased functional activity, as determined by enriched pathways with an adjusted *P* value of <0.05. No significantly downregulated pathways were observed. (c) Heat map showing DEGs involved in RNA processing and mitochondrial complexes that are upregulated under ADE conditions. For all analyses, DEGs were identified based on a fold change in expression of ≥1.5 compared to the level of expression by mock-infected cells with an FDR *q* value of ≤0.1. Only GO terms with an adjusted *P* value of ≤0.05 are shown.

To identify the biological processes that are influenced by ADE, we performed Gene Ontology (GO) term analysis of the DEGs. The most enriched upregulated biological processes were found to be related to RNA splicing and mitochondrial respiratory chain complexes (Fig. 3a). These DEGs reside mostly in the spliceosomal complexes, nuclear bodies, and mitochondria (Fig. 3b; Fig. S3). The DEGs related to RNA processing were uniquely upregulated under ADE conditions (Fig. 3c). Notably, several of the genes that were uniquely upregulated with ADE, such as small nuclear ribonucleoprotein polypeptide F (SNRPF), pre-mRNA processing factor 19 (PRPF19), heterogeneous nuclear ribonucleoprotein A1 (HNRNPA1), and HNRNPU (Fig. 3c), have also previously been shown to be DENV host factors that interact with DENV RNA (36, 37, 39).

10.1128/mSphere.00528-19.3 FIG S3 Spliceosomal genes are upregulated under ADE conditions. Genes (highlighted in red) associated with the spliceosome that are significantly upregulated under ADE conditions. Genes in purple are not differentially expressed under ADE conditions. KEGG pathway hsa03040 is shown. Download FIG S3, TIF file, 2.8 MB. Copyright © 2019 Chan et al. 2019 Chan et al. This content is distributed under the terms of the Creative Commons Attribution 4.0 International license.

In addition, components of the mitochondrial respiratory chain complexes, especially complex I, were also upregulated under ADE conditions. While a subset of these genes, such as mitochondrial ND1 (MT-ND1), MT-ND2, and MT-ND4, was significantly upregulated under both ADE and HI-ADE conditions, other mitochondrial genes, such as MT-ND2P13, NDUFA10, NDUFS5, ATP5C1, MT-ATP6, and MT-CYB, were induced only under ADE conditions (Fig. 3c). No significantly downregulated enriched pathways were detected under ADE conditions. Interestingly, and unlike the findings obtained under ADE conditions, no significantly enriched pathways were observed under HI-ADE conditions. These findings collectively suggest that early virus trafficking and compartmentalization from activating FcγR-mediated entry and internalization drive the upregulation of host transcripts related to the assembly of macromolecules, such as spliceosomes and mitochondrial respiratory chain complexes, to favor DENV replication.

### ADE impeded the downregulation of host ribosomal genes caused by canonical receptor-mediated entry.

**FIG 4:**
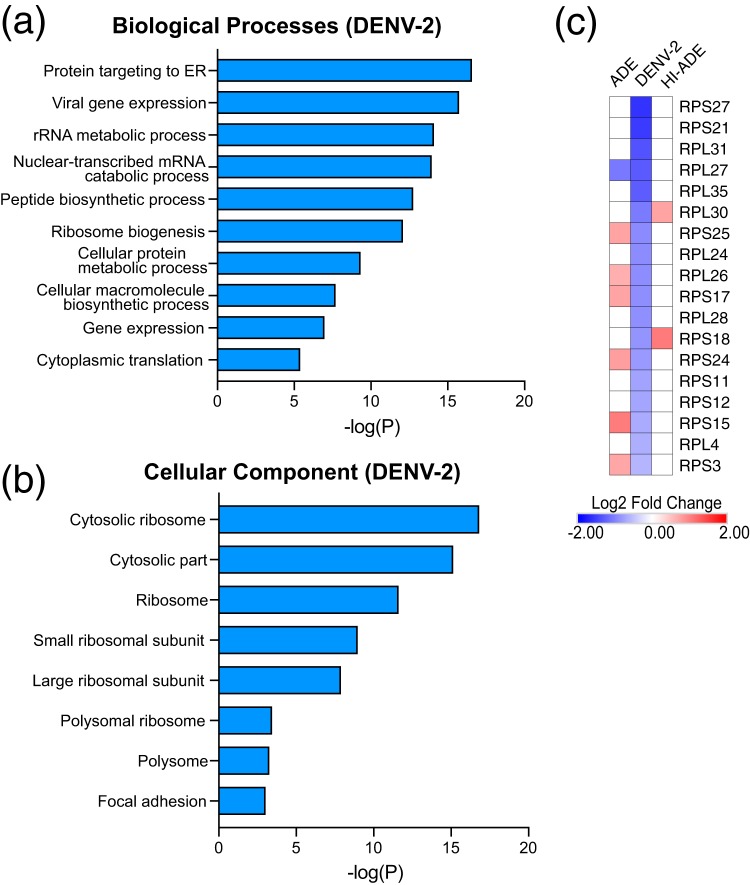
ADE-mediated DENV entry impedes the downregulation of ribosomal genes caused by canonical receptor-mediated endocytosis. (a and b) Gene Ontology (GO) annotation of differentially expressed genes (DEGs) at 6 hpi after exposure to unopsonized DENV, classified by biological processes (a) and cellular component (b), as determined using EnrichR analysis. Blue bars indicate significantly decreased functional activity, as determined by enriched pathways with an adjusted *P* value of <0.05. No significantly upregulated pathways were observed. (c) Heat map showing ribosomal DEGs that are downregulated under DENV conditions. For all analyses, DEGs were identified based on a fold change in expression of ≥1.5 compared to the level of expression by mock-infected cells with an FDR *q* value of ≤0.1. Only GO terms with adjusted *P* values of ≤0.05 are shown.

We next investigated the transcriptomic profiles that were uniquely altered by the DENV cell entry pathway. Canonical receptor-mediated entry resulted in more downregulated DEGs than upregulated DEGs (Fig. 2b and c). The significantly enriched downregulated pathways were mostly related to protein translation (Fig. 4a). The cellular compartment subontology further revealed that a large proportion of these downregulated genes were ribosomal proteins, including the large and small subunits (Fig. 4b; Fig. S4). This finding is consistent with the findings of previous studies showing the downregulation of genes related to translation in the blood of patients who have been acutely infected with dengue virus (40). Moreover, these observations support data from Flipse et al. showing an increase in viral translation under ADE conditions compared to DENV-only infection (23). Given the requirement for host ribosomes in the endoplasmic reticulum (ER) for DENV RNA translation (41), our results suggest the possibility that ADE-mediated entry impedes the downregulation of ribosomal genes caused by canonical receptor-mediated entry, thereby promoting viral protein translation.

10.1128/mSphere.00528-19.4 FIG S4 Ribosomal genes are downregulated under DENV infection conditions. Genes (highlighted in blue) associated with the ribosome that are significantly downregulated under DENV infection conditions. KEGG pathway hsa03040 is shown. The other ribosomal genes expressed in eukaryotes are annotated in green, but these are not differentially expressed under DENV infection conditions. KEGG pathway hsa03040 is shown. Download FIG S4, EPS file, 2.6 MB. Copyright © 2019 Chan et al. 2019 Chan et al. This content is distributed under the terms of the Creative Commons Attribution 4.0 International license.

### ADE increased the expression of DENV host dependency factors.

To investigate how ADE-mediated entry can modulate the transcriptome of monocytes to favor virus infection, we cross-referenced all DEGs modulated by DENV and ADE-mediated viral entry with recent genome-wide small interfering RNA (siRNA) and CRISPR/Cas9 screens (33–37, 42). Compared to the other conditions, ADE induced the highest number of upregulated viral dependency genes (Table 1), including genes related to mRNA processing (HNRNPU, SNRPF, SF3B1, SUPT6H) and vesicle-mediated transport (SCFD1, PREB). Only 2 host dependency factors, one encoding zinc transporter SLC39A11 and the other encoding endocytic adaptor protein NUMBL, were upregulated under both DENV and ADE conditions (Table 1). The only host dependency factor that was downregulated under ADE conditions was the osteoclast-associated receptor (OSCAR) gene, which has been shown to be physically associated with FcγR to mediate endocytosis and proinflammatory responses (43). In sharp contrast, canonical receptor-mediated entry upregulated fewer viral dependency factors than ADE. Instead, it downregulated the highest number of viral dependency factors among the other conditions, many of which belong to ribosomal transcripts that are involved in viral protein translation (Table 2). Taken together, our results show that ADE augments the expression of DENV host dependency factors to create an intracellular environment that is conducive for viral replication, translation, and trafficking.

**TABLE 1:**
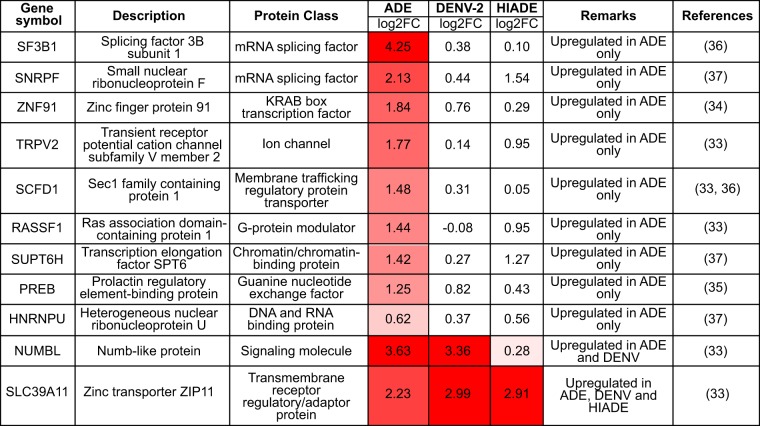
Upregulated host dependency factors under ADE, DENV2, or HI-ADE conditions compared to mock-infected conditions^*a*^

a Log_2_ fold change (log2FC) values are shown. The significantly upregulated genes are depicted in the “Remarks” column, where only upregulated DEGs with a fold change in expression of >1.5 compared to the level of expression by mock-infected cells, a *P* value of <0.05, and a *q* value of <0.1 are highlighted in red. The exact *P* and *q* values are indicated in Table S1 in the supplemental material. Red indicates upregulation with respect to mock-infected cells. Darker shades correspond to upregulated fold changes with greater magnitude.

**TABLE 2:**
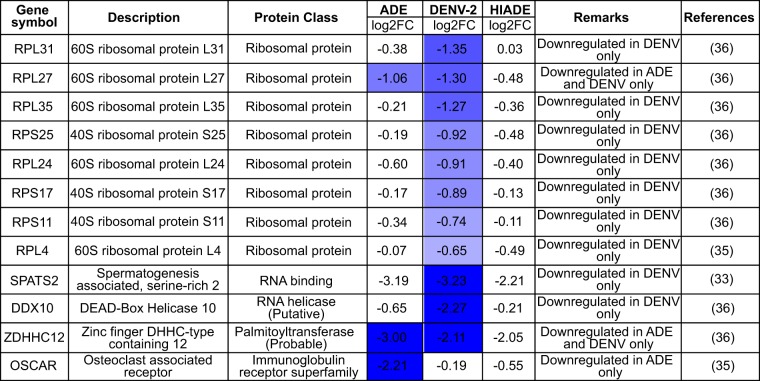
Downregulated host dependency factors under ADE, DENV2, or HI-ADE conditions compared to mock-infected conditions^*a*^

a Log_2_ fold change (log2FC) values are shown. The significantly downregulated genes are depicted in the “Remarks” column, where only downregulated DEGs with a fold change in expression of >1.5 compared to the level of expression by mock-infected cells, a *P* value of <0.05, and a *q* value of <0.1 are highlighted in blue. The exact *P* and *q* values are indicated in Table S1 in the supplemental material. Blue indicates downregulation with respect to mock-infected cells. Darker shades correspond to downregulated fold changes with greater magnitude.

### Transcriptional responses altered by ADE.

**FIG 5:**
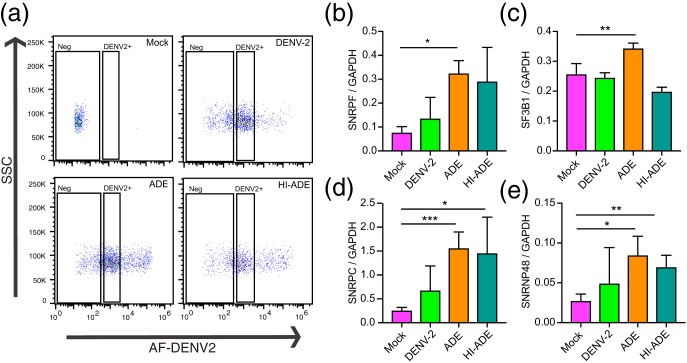
Differential expression of spliceosomal genes under ADE conditions. (a) Representative flow cytometry plots for mock-infected, DENV2, ADE, and HI-ADE conditions mediated by polyclonal antibodies from convalescent-phase serum after 6 hpi. Cells within a restricted range of fluorescence intensity (DENV2^+^) were sorted by FACS for further transcriptomic analysis. (b to e) Transcript levels of SNRPF (b), SF3B1 (c), SNRPC (d), and SNRNP48 (d) in DENV2^+^ cells infected under DENV2, ADE, or HI-ADE conditions, measured by quantitative PCR. Data are expressed as the mean ± SD from four independent experiments. ***, *P* < 0.001; **, *P* < 0.01; *, *P* < 0.05.

We next ascertained if the observed differences in transcriptional profiles can be reproduced in DENV opsonized with polyclonal antibodies. DENV2 was first preincubated with convalescent-phase serum, and the resultant immune complexes were used to infect primary monocytes. At the peak enhancement of infection (Fig. S5), the uptake of AF488-DENV2 was increased compared to that with unopsonized infection (Fig. 5a). Using the same gating strategy, cells with similar antigenic loads under DENV, ADE, and HI-ADE conditions were sorted (Fig. 5a). Consistent with h3H5-mediated internalization, ADE mediated by polyclonal anti-dengue virus serum significantly upregulated the transcript expression of spliceosome genes, including SNRF, SF3B1, SNRPC, and SNRNP48, as determined by RT-qPCR (Fig. 5b to e). Notably, SNRF and SF3B1, which have been found to be host dependency factors for DENV (36, 37), were uniquely upregulated under ADE conditions but not under DENV2 or HI-ADE conditions. Collectively, these findings support the notion that ADE-mediated viral entry induces host transcriptional responses that are distinct from those induced by canonical receptor-mediated endocytosis.

10.1128/mSphere.00528-19.5 FIG S5 Enhancement of infection mediated by convalescent-phase serum. Plaque titers of primary monocytes when infected with DENV2 opsonized with different dilutions of convalescent-phase serum at 72 h postinfection. Peak enhancement was observed at a titer of 1:640 (indicated with a red arrow), and this titer was used for subsequent ADE assays. The dashed line indicates plaque titers in the presence of DENV2 only, without antibody opsonization. Data are represented as the mean ± SD from three independent experiments. **, *P* < 0.01. Download FIG S5, EPS file, 0.8 MB. Copyright © 2019 Chan et al. 2019 Chan et al. This content is distributed under the terms of the Creative Commons Attribution 4.0 International license.

## DISCUSSION

Antibody-mediated cell entry of DENV has been shown to facilitate a more efficient entry into the target cells of DENV, myeloid-derived monocytes, macrophages, and dendritic cells, than infection in the absence of enhancing antibodies. The more efficient route of entry has often been attributed to be the cause of enhancement of DENV infection. Others have postulated that besides a more efficient route of viral entry, activating FcγR-mediated entry could also result in a suppression of type I interferon or interferon-activated antiviral molecules at the later stages of infection (4, 5, 24, 25, 28). However, as DENV infection in myeloid cells is more efficient under ADE conditions, the extent to which genes are regulated by differential receptor engagement or antigenic load remains to be determined. This study was thus directed at controlling for the viral antigenic load in order to tease apart the host response that is either induced by the entry pathway or dependent on the viral antigenic load.

When the antigenic load is controlled, genes associated with RNA splicing and mitochondrial respiratory complexes were found to be the most upregulated under ADE conditions. In contrast, canonical receptor-mediated viral entry resulted in the downregulation of ribosomal proteins that have previously been shown to be critical for viral and host protein translation (39). These contrasting transcriptomic changes were observed despite the presence of similar levels of internalized DENV particles, suggesting that the pathway of viral entry can impact the host responses to DENV. Furthermore, by combining our transcriptomic findings with published data sets on flavivirus host dependency factors, we identified the host dependency factors were induced in primary monocytes but only under ADE conditions. These upregulated factors are involved in various stages of the DENV life cycle, including DENV RNA binding proteins, which are required for efficient DENV amplification (36, 39); ER-Golgi apparatus trafficking proteins, which are required for virus transport; and mitochondrial complexes, which are required for ATP synthesis (34, 36, 39). As we found that ADE increases spliceosomal genes, it is also plausible that ADE may modify the splicing landscape of the infected cells to generate an intracellular environment more favorable for viral replication and packaging. However, determination of the precise mechanisms by which ADE alters different splicing isoform abundance requires further investigations that were beyond the scope of and resources available for this study. Nonetheless, these identified upregulated host dependency factors that are preferentially modulated by antibody-mediated DENV uptake could be potential targets for the development of therapeutics against severe dengue.

On the other hand, canonical receptor-mediated endocytosis resulted in the downregulation of ribosomal genes, which can affect host and viral translation. This result is similar to that of studies showing that ribosomal genes are downregulated in the whole blood of patients that have been acutely infected with dengue virus (40). While the mechanisms have not been clearly defined, it is plausible that the downregulation of ribosomes is a part of the response to ER stress (44, 45). If this is true, it would suggest that ADE enables DENV to manage ER stress to favor successful completion of its life cycle; we have previously found that ER stress inhibits DENV egress through exocytosis (46). It is also possible that the induction of mitochondrial genes under ADE conditions may aid in providing sufficient ATP, required for stress adaptation (47). The consequent higher expression of ribosomal genes under ADE conditions may thus explain the increased viral translation under ADE conditions compared to DENV-only infection conditions (23). Our finding of reduced expression of host dependency factors thus suggests that increasing the inoculum of DENV during infection without enhancing antibodies may be paradoxically counterproductive for DENV replication.

Interestingly, we also observed few similarities between the transcriptomic profiles of ADE- and HI-ADE-mediated uptake, even though both conditions likely and similarly engage FcγR and LILRB1. This finding underscores the notion that the cellular response to flavivirus infection is a complex multifactorial process. For instance, flavivirus replication results in the production of viral proteins that antagonize host cell antiviral signal transduction and alter gene expression to favor replication and spread (48–53). The loss of the replicative ability of inactivated DENV may thus account for the observed differences. Another possible explanation is that during heat inactivation, some of the viral lipid complexes and glycoproteins become denatured. This denaturation could in turn disrupt the integrity of attachment sites to hitherto unidentified cell signaling proteins, thus modifying gene expression and even intracellular trafficking, which in turn alters the host gene expression profile. Finally, it is also possible that the differential intracellular rate of fusion or compartmentalization under ADE, DENV, and HI-ADE conditions could lead to exposure of the virus to a different repertoire of vesicular receptors, including pathogen recognition receptors, resulting in differential cellular responses (20, 47, 48, 54, 55). Future studies directed at elucidating how the differential compartmentalization of DENV can influence receptor interactions and host responses could bring novel insights for dengue pathogenesis.

Our findings indicate that besides facilitating DENV entry, antibody-dependent DENV entry upregulated a list of host dependency genes that are known to support DENV infection. Hence, in addition to polymorphisms and baseline expression levels of activating FcγRs, variations in the expression of host dependency genes that are differentially modulated by ADE may also influence host susceptibility to ADE. Follow-up experiments that systematically explore the contribution of these genes in ADE-mediated DENV infection could thus provide deeper insights into the genetic determinants for severe dengue.

In conclusion, our findings demonstrate that the host response to DENV is influenced by the portal of viral entry. The host dependency factors specifically modulated by FcγR-mediated DENV entry identified here represent attractive targets for the development of therapeutics for severe dengue.

## MATERIALS AND METHODS

### Cells.

THP-1, Vero, and BHK-21 cells were purchased from ATCC and cultured according to ATCC’s recommendations. Human primary monocytes from a dengue virus-naive healthy donor were collected (with the approval of the NUS Institutional Review Board under reference code B-15-227) in BD sodium heparin Vacutainer tubes (Biomed Diagnostics). The blood was then diluted with 2 volumes of 0.5% bovine serum albumin (BSA; Sigma-Aldrich) in phosphate-buffered saline (PBS; 1st base) (0.5% BSA–PBS) and then slowly layered onto Ficoll-Hypaque at room temperature (GE Healthcare). The blood was then centrifuged at 750 × *g* without the use of brakes. The interphase cells containing the peripheral blood mononuclear cells (PBMCs) were aspirated and transferred to a clean tube. The PBMCs were washed three times with 0.5% BSA–PBS and resuspended in growth medium (RPMI 1640 supplemented with 10% fetal bovine serum albumin, 100 U/ml penicillin, 100 μg/ml streptomycin). Cells were counted and then seeded into T75 tissue culture flasks (Nunc; Bio Laboratories) at 3 × 10^7^ to 5 × 10^7^ cells/flask and incubated at 37°C in 5% CO_2_ for 2.5 h to allow the monocytes to adhere to the flask surface. The cells were washed with PBS to remove the nonadherent lymphocytes, and fresh growth medium was added. Monocytes were incubated overnight at 37°C in 5% CO_2_ before further use in experiments.

### Fluorescent labeling of DENV.

DENV2 (ST) is a clinical isolate from Singapore General Hospital. It was propagated in Vero cells and purified through 30% sucrose. The titer of stock virus was determined by plaque assay on BHK-21 cells as previously described (56). For Alexa Fluor 488 (AF488) dye-labeled viruses, purified DENV2 (ST) was labeled and quantified as previously described (38).

### Heat inactivation of virus.

To heat inactivate the viruses, AF488-labeled DENV2 (AF488-DENV2) aliquots were incubated in a 56°C water bath for 30 min and gently mixed every 15 min. The noninfectivity of heat-inactivated virus was confirmed by plaque assay, as previously described (38).

### DENV2 infection in monocytes.

For ADE infection, chimeric human/mouse IgG1 3H5 (h3H5) monoclonal antibodies were used and produced as previously described (57). h3H5 (0.39 μg/ml), which has previously been shown to result in maximal ADE in primary monocytes (58), was incubated with AF488-DENV2 or heat-inactivated AF488-DENV2 for 1 h at 37°C. For opsonization with polyclonal serum, we utilized an anti-DENV convalescent-phase serum that cross-reacts with all 4 DENV serotypes, as determined by a plaque reduction or neutralization test. Immune complexes are formed with enhancing concentrations of multitypic polyclonal serum (1:640), as determined by plaque assay (24, 56). Thereafter, DENV or DENV immune complexes were added to primary monocytes at an MOI of 10, and the cells were incubated for 6 h and 22 h at 37°C. After washing the cells thrice with PBS, the cells were then resuspended in PBS with 1% fetal calf serum before subjecting the cells to FACS. h3H5 alone at 0.39 μg/ml or polyclonal serum at a 1:640 dilution served as a control.

### Immunofluorescence.

THP-1 cells were incubated with AF488-DENV2, h3H5-opsonized AF488-DENV2, or heat-inactivated AF488-DENV at 37°C for 6 h. After washing the cells thrice with PBS, the cells were fixed with 3% paraformaldehyde for 30 min on ice. Thereafter, the cells were subjected to a cytospin at 800 × *g* for 3 min. After washing with PBS, the cell membrane was labeled with anti-CD32 (diluted in permeabilization buffer containing 0.1% saponin in PBS, 5% BSA, 0.2% gelatin) for 45 min at room temperature. Subsequently, the cells were washed with PBS thrice and incubated with anti-mouse immunoglobulin secondary AF594-conjugated antibody and DAPI (4′,6-diamidino-2-phenylindole) dye diluted in permeabilization buffer for 45 min at room temperature. After extensive washing with PBS, the cells were subjected to washing with PBS and mounted on slides with Mowiol mucoadhesive. Samples were then viewed under a laser-scanning confocal microscope (Leica TCS SP2).

### ELISA.

Live or heat-inactivated DENV2 was coated on a MaxiSorp plate and incubated with soluble LILRB1 ectodomain as previously described (24).

### Nanoparticle tracking analysis.

Live or heat-inactivated DENV2 was suspended in sterile PBS. For Triton X-100 treatment, DENV2 was resuspended with 10% Triton X-100 (Sigma-Aldrich) diluted in PBS, followed by vortexing for 30 s and incubation at room temperature for 1 h. For Milli-Q water treatment, DENV2 was resuspended in Milli-Q water in a 1:1 volume, followed by vortexing for 30 s and incubation at room temperature for 1 h. All viruses were then further diluted in filtered HNE buffer (5 mM HEPES, 150 mM NaCl, 0.1 mM EDTA, pH 7.4) before taking nanoparticle tracking analysis measurements with a NanoSight LM10 instrument (Malvern Instruments, United Kingdom) at room temperature.

### FACS.

Fluorescence-activated cell sorting (FACS) experiments were performed on a BD FACSAria III flow cytometer. Mock-infected monocytes were used to define the negative gate. For monocytes infected with AF488-DENV2, h3H5-opsonized AF488-DENV2, or polyclonal serum-opsonized AF488-DENV2, a positive gate was used to define the infected cell population, and only a subset of cells within a restricted range of mean fluorescence intensity was sorted for further analysis. A minimum of 5,000 cells were sorted directly into RNeasy RLT lysis buffer (Qiagen) and stored at −80°C.

### Generation of whole-transcriptome cDNA library.

RNA was extracted using an RNeasy microkit (Qiagen) per the manufacturer’s instructions and assessed by electrophoresis on a Bioanalyzer 2100 instrument (Agilent Technologies) for quality evaluation. For sample preparation for transcriptome sequencing (RNAseq), an Ovation single cell RNAseq system (Nugen) was used according to the manufacturer’s protocol. Briefly, 10 ng of total RNA was subjected to DNase treatment and first-strand cDNA synthesis using whole-transcriptome primers that target non-rRNA sequences. The cDNA was then fragmented to an average size of 230 nucleotides and subsequently ligated to paired-end adaptors to produce strand-specific libraries. Finally, the library was enriched using 12 cycles of PCR and purified by the use of Agencourt RNAClean XP beads (Nugen).

### RNAseq analysis.

RNA sequencing was performed at the Duke-NUS Genome Biology Core Facility on Illumina MiSeq and Illumina HiSeq 2000 machines. The MiSeq run was performed with the paired-end 250-bp-read option, and the HiSeq run was performed with the paired-end 100-bp-read option. The quality of the paired sequencing reads was verified by use of the FastQC tool. For the HiSeq reads, the average sequencing depth was 16.5 million reads per sample and the per sequence quality score was ≥37 for all the reads. No further trimming of the bases was performed for the HiSeq reads. The sequencing reads were mapped to the human reference genome GrCH38 (Gencode release 24) using the TopHat alignment tool (59). The mean mapping rate was 81.17%. Transcript/gene assembly and abundance analysis were performed with the Cufflinks program to generate counts normalized for transcript length and library size, given as the number of fragments per kilobase of transcript per million mapped reads (FPKM). The expression values under the DENV-only, ADE, and HI-ADE conditions were compared to the expression values for mock-treated monocytes to calculate the expression fold change, *P* values, and FDR *q* values. Genes with *q* values of <0.1, *P* values of <0.05, and a fold change in expression of >1.5 or ≤1.5 compared to the level of expression by mock-infected cells were considered differentially expressed genes (DEGs), and the list of genes was used as the input for further pathway analysis. PCA was performed on the genes with FPKM values of more than 5 and constructed using the R Studio program. The packages used included factoextra (60), ggplot2 (61), and FactoMineR (62). To identify the pathways that were significantly altered by the route of infection, the upregulated or downregulated DEGs were subjected to EnrichR analysis (63). The adjusted *P* values for the enriched pathways (adjusted *P* value < 0.05) in GO biological processes and GO cellular component were determined by EnrichR analysis. The specific genes related to spliceosome and ribosomes were mapped into the KEGG database (64).

### DENV and gene transcript measurements in infected cells.

DENV and gene transcript levels in the DENV2^+^ cells sorted by FACS were measured by RT-qPCR as previously described (56). Briefly, the sorted cells were resuspended in RLT buffer with beta-mercaptoethanol. After extraction of RNA using the RNeasy microkit (Qiagen), cDNA synthesis (Quanta) and real-time qPCR (Roche) were performed with SNRPF, SF3B1, SNRPC, and SNRNP48 primers (PrimerBank), using GAPDH (glyceraldehyde-3-phosphate dehydrogenase) as the housekeeping gene. The specific sequences are listed in Table S2 in the supplemental material.

10.1128/mSphere.00528-19.7 TABLE S2 Primer sequences used for the qPCR validation. Download Table S2, XLSX file, 0.01 MB. Copyright © 2019 Chan et al. 2019 Chan et al. This content is distributed under the terms of the Creative Commons Attribution 4.0 International license.

### Statistical analysis.

A two-tailed unpaired Student’s *t* test was used to determine if the difference in the mean observed was statistically significant (*P* < 0.05). All calculations were performed with GraphPad Prism (v8.1.0) software (GraphPad Software Inc.).
