# Supplementary figures and images for: Antibody-Dependent Dengue Virus Entry Modulates Cell Intrinsic Responses for Enhanced Infection
Source: mSphere. 2019 Sep 18;4(5):e00528-19. doi: 10.1128/mSphere.00528-19 (PMC6751492; doi:10.1128/mSphere.00528-19)

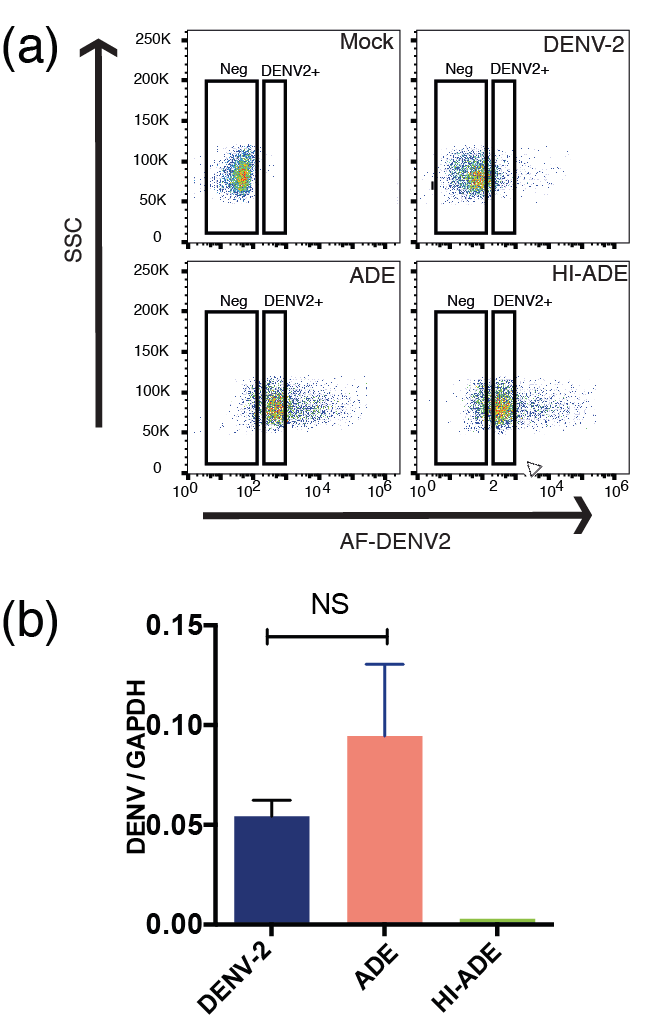

Supplement: FIG S1 [file mSphere.00528-19-sf001.tif]

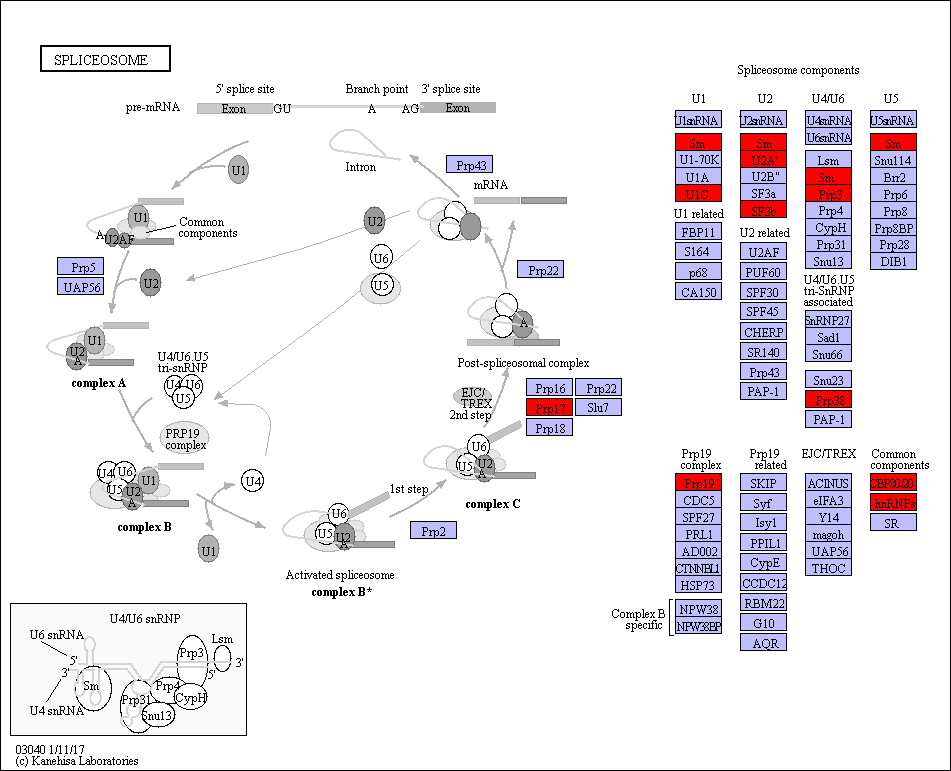

Supplement: FIG S3 [file mSphere.00528-19-sf003.tif]
